# Effect of Biostimulant, Manure Stabilizer, and Manure on Soil Physical Properties and Vegetation Status

**DOI:** 10.3390/plants13070920

**Published:** 2024-03-22

**Authors:** Václav Novák, Petr Šařec, Oldřich Látal

**Affiliations:** 1 Department of Machinery Utilization, Faculty of Engineering, Czech University of Life Sciences Prague, 165 00 Prague, Czech Republic; novakvaclav@tf.czu.cz; 2 Agrovyzkum Rapotin Ltd., Vyzkumniku 863, 788 13 Rapotin, Czech Republic; latal.oldrich@post.cz

**Keywords:** bulk density, cone index, crude fat, crude protein, NeOsol, saturated hydraulic conductivity, unit draft, remote sensing, yield, Z’fix

## Abstract

Food production sustainability is one of contemporary agriculture’s fundamental challenges. Farmers are currently facing high input prices in crop production and declining organic matter in the soil. For this reason, a field experiment was established to assess the effect of the biostimulant NeOsol (NS), the manure stabilizer Z’fix (ZF), farmyard manure (FM), and their combination in farm practice. In situ measurements provided information on the change in bulk density (BD), unit draft (UD), saturated hydraulic conductivity (SHC), and cone index (CI). Furthermore, the vegetation status was investigated via vegetation indices, and the yield and quality parameters were assessed. Management of the experimental field resulted in an overall decrease in BD over time for the treated variants compared to the control (CL). The decrease with time was also verified in the case of UD and CI at the depth zone of 10–20 cm. Variants FM (by 8.0%), FM_NS (by 7.3%), and FM_ZF_NS (by 3.8%) proved to have lower UD values than CL. An overall increase in SHC and in yield was observed over time. Concerning SHC, only FM (by 58.5%) proved different from CL. The yield of all the treated variants, i.e., NS (by 8.2%), FM (by 10.8%), FM_NS (by 14.1%), FM_ZF (by 17.8%), and FM_ZF_NS (by 20.1%), surpassed CL. Simultaneously, none of the examined treatments proved to have any adverse effect either on soil or on plant-related variables.

## 1. Introduction

Contemporary agriculture is facing demanding challenges unprecedented in history. Currently, there are eight billion people in the world. The United Nations estimates there will be over 8.5 billion people by 2030 and even more than 11 billion by 2050 [1]. With this population growth, a decrease of 50 million hectares of arable land in developed countries is also expected [2]. Regarding the Green Deal, a decline in livestock production of 10 to 15 percent can be expected [3].

With the anticipated decline in livestock farming, there will also be a decline in farmyard manure production and availability. While farmyard manure has a positive effect on soil fertility and consequently on plant yields, synthetic fertilizers provide large amounts of nutrients to the soil—and hence increase yields—but reduce the carbon content of the soil and do not improve soil health [4]. In terms of sustainability, it is appropriate to integrate plant-based products—such as algae or microorganism-based products—into agricultural practice [5,6]. Some companies may add value to products, e.g., stranded seaweed on beaches, thus facilitating the restoration of ecosystems. This is the case with the French group Olmix [7].

The company produces diverse biostimulants, soil improvers, agents, and stabilizers; this study investigated the following two: NeOsol (formerly known as PRP SOL) and Z’fix (formerly known as PRP FIX). According to the manufacturer, (a) NeOsol stimulates the natural soil microflora, which is involved in the recycling and transforming of crop residues. By supporting humus synthesis, NeOsol enhances the components of soil fertility and soil health. (b) Z’fix reduces ammonia emissions from stable bedding or effluents and moreover improves effluents’ fluidity and homogeneity. This simplifies spreading work and assists in the preservation of nutrients for the crops.

Several studies have been conducted to explore the efficacy of these amendments: (a) Findura et al. [8] observed over a three-year period that biostimulants positively influenced crop yield and soil bulk density and increased oxidizable carbon. Similarly, Šindelková et al. [9] reported a favourable effect on soil physical and chemical properties in a short-term experiment. In addition, the biostimulant improved the C/N ratio [10]. Subsequent short-term research indicated improvements in water retention [11]. The two-year results of the study by Tuba et al. [12] showed a reduction in cone index and also in the energy demand for ploughing. The authors recommended long-term use of the product for cumulative positive effects. A long-term study (10 years) of NeOsol investigated its effect on the chemical composition of soils. Soil pH increased and the content of available forms of magnesium increased; on the other hand, there was a decrease in available forms of potassium [13]. In a two-year study, a biostimulant significantly increased the chlorophyll content of rye leaf blades [14]. NeOsol combined with urea increased soybean yield [15]. The positive effect of the product was also demonstrated in potatoes, where the highest greenness index was observed [16]. The product improved the growth and yield of calendula [17].

(b) The results of the three-year experiment proved that manure treated with Z’fix positively affected the soil conditions and crop status [18]. Slurry stabilizer treatment resulted in higher macronutrient content in the soil [19]. This agent was found to positively influence the fermentation process and the manure quality [20]. Plants usually contained more nitrogen, phosphorus, potassium, magnesium, calcium, and sulphur when treated slurry had been applied [19]. Higher nitrogen content was achieved also in another study [21].

Even with the increasing use of biostimulants in agricultural practice, many scientific community members perceive that biostimulants need peer-reviewed scientific evaluation [22]. The majority of the results described above were based on short-term experiments.

This study aims to verify the effect of the biostimulant, the stabilizer, manure, and their combinations in real conditions of agricultural practice in a long-term experiment carried out on Modal Luvisol of the silt loam soil texture. The hypotheses to be verified are as follows: the use of these improvers would result in (a) a reduction in the unit draft, soil bulk density, and cone index, (b) an increase in saturated hydraulic conductivity, (c) an improvement in crop growth, and (d) an increase in crop yield and improved qualitative parameters.

## 2. Results

### Soil Physical Characteristics

Variables describing soil physical characteristics were assessed in three years, i.e., terms (see Materials and Methods), following autumn manure application. Figures present their values relative to the value of the respective control variant (CL) since, in addition to the variants, there may have been other factors affecting the variables’ absolute values, e.g., soil moisture and machinery passes. These factors can be considered though to have had the same effect on all the variants including CL. The tables contain the absolute values with significant differences marked within individual terms. Within individual terms, one-way analyses of variance (ANOVA) provides the same outcome for the absolute values as well as for the relative ones. When using factorial (variant; term) ANOVA, the outcomes of analysis differ depending on whether absolute or relative values are compared. If the latter ones are used, the differences among terms will provide information on the variable development relative to CL.

**Figure 1:**
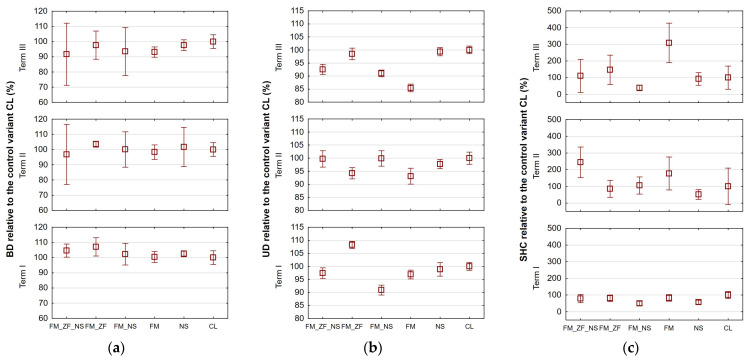
Values of soil physical characteristics: (**a**) bulk density (BD); (**b**) unit draft (UD); and (**c**) saturated hydraulic conductivity (SHC) relative to the respective control variant (CL) for different variants of farmyard manure (FM), farmyard manure stabilized by Z’fix (FM_ZF), and biostimulant NeOsol (NS) application in individual terms; vertical bars denote 0.95 confidence intervals.

The bulk density (BD) values related to the CL variant are shown in Figure 1a. Table 1 presents the absolute values and their statistical evaluation analysis for each term. Factorial ANOVA of relative values proved that Term III (95.609%) was significantly different compared to Term II (100.075%) and Term I (102.835%). This suggested a positive effect of the treatments over a longer period of time. Factorial ANOVA of absolute values proved that Term I (1.491 g cm^−3^) was significantly different compared to Term II (1.328 g cm^−3^) and Term III (1.353 g cm^−3^). One-way ANOVA proved that there were significant differences among variants solely in Term I, when standard deviations demonstrated the lowest values. The BD of CL and FM variants attained significantly lower values than that of FM_ZF (see Table 1).

Figure 1b presents the unit draft (UD) values of the variants relative to CL. The factorial ANOVA proved that individual terms significantly differed among themselves, with Term I showing the highest overall value of 99.454%, Term II showing a value of 97.439%, and Term III showing the lowest value of 94.629%. Factorial ANOVA of absolute values proved also significantly different among terms. In that case, the differences should be attributed to other varying factors rather than to the effect of variants, such as soil moisture, working depth, working speed, preceding crop, etc. One-way ANOVA proved that there were significant differences among variants within all three terms. In Term I (see Table 1), variants formed three significantly differing homogenous groups, with FM_ZF forming that with the highest UD and FM_NS forming the group with the lowest UD. In Term II, the average UD of the variants CL, FM_NS, and FM_ZF_NS significantly exceeded the UD of FM and FM_ZF. In Term III, variants formed again three significantly differing homogenous groups with the variants CL, NS, and FM_ZF forming that with the highest UD and FM forming the group with the lowest UD. Based on the analyses described, there does not seem to be any pattern discernible. When performing factorial ANOVA of relative values combined for all three terms though, variants form four homogenous groups with an apparent pattern, where only CL belongs simultaneously to two groups. The group with the highest UD values consists of FM_ZF (101.422%) and CL (100.000%). Then, a group of CL and NS (98.758%) follows, trailed by a group comprising a single variant, FM_ZF_NS (96.231%). Finally, the group with the lowest UD is formed by FM_NS (93.726%) and FM (92.047%).

Saturated hydraulic conductivity (SHC) values relative to the control variant CL are presented in Figure 1c. Factorial ANOVA of relative values proved that Term I (74.658%) was significantly different compared to Term II (127.622%) and Term III (134.559%). This result suggests that the SHC of the treated variants improved over time relative to the control variant. Factorial ANOVA of absolute values proved that there were significant differences among all three terms, with Term I showing the lowest overall value of 5.038 mm h^−1^, Term III showing a value of 22.982 mm h^−1^, and Term II showing the highest value of 37.718 mm h^−1^. One-way ANOVA proved that there were significant differences among variants within all three terms. In Term I (see Table 1), the SHC of CL attained a significantly higher average than those of NS and FM_NS. In Term II, the SHC of FM_ZF_NS attained a significantly higher value than all the other variants except FM. Finally, in Term III, it was the FM variant that demonstrated a significantly higher SHC than all the other variants.

**Figure 2:**
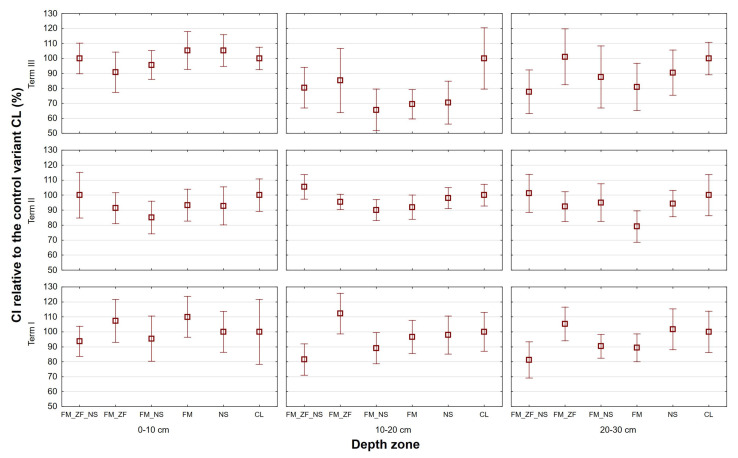
Values of cone index (CI) for different variants of farmyard manure (FM), farmyard manure stabilized by Z’fix (FM_ZF), and biostimulant NeOsol (NS) application in individual terms; vertical bars denote 0.95 confidence intervals.

Figure 2 presents the cone index (CI) averages of the variants relative to CL at three soil depth zones of 0 to 10 cm, 10 to 20 cm, and 20 to 30 cm. Their factorial ANOVA proved that there was a significant difference among terms only at the middle zone of 10 to 20 cm, with Term III differing the most with its lowest overall value of 78.742%when compared to the Term I value of 96.234% and the Term II value of 96.825%. With respect to the variants, the factorial ANOVA of relative values found significant differences at the depth zones of 10 to 20 cm and 20 to 30 cm. At the 10 to 20 cm depth, the CI average of FM_NS (82.096%) significantly differed from FM_ZF (97.656%) and from CL (100.000%); the CI average of FM (85.989%) also significantly differed from CL (100.000%). At the 20 to 30 cm depth, the CI average of FM (83.136%) significantly differed from FM_ZF (99.590%) and from CL (100.000%). Factorial ANOVA of absolute values proved that there were significant differences among terms at all three depth zones. At the 0 to 10 cm depth, the CI averages differed significantly among all three terms, with Term III demonstrating the lowest value (0.378 MPa), followed by Term I (0.558 MPa) and, finally, Term II (0.975 MPa). At the 10 to 20 cm depth, the CI averages differed significantly among Term III (again, the lowest value was 0.711 MPa) and both Term I (1.232 MPa) and Term II (1.288 MPa). At the 20 to 30 cm depth, the CI averages differed significantly again among all three terms, with Term III demonstrating the lowest value (1.650 MPa), followed by Term II (1.902 MPa) and, finally, Term I (2.314 MPa). With respect to the variants, the factorial ANOVA of absolute values proved that there were significant differences at the depth zones of 10 to 20 cm and 20 to 30 cm that presented a similar pattern to the factorial ANOVA of relative values. At the 10 to 20 cm depth, the CI average of FM_NS (0.990 MPa) significantly differed from FM_ZF (1.159 MPa) and from CL (1.171 MPa). At the 20 to 30 cm depth, the CI average of FM (1.760 MPa) significantly differed from FM_ZF (2.103 MPa) and from CL (2.105 MPa).

One-way ANOVA proved that there were significant differences among variants only at the soil depth zones of 10 to 20 cm and 20 to 30 cm (see Table 2). At the 10 to 20 cm depth, the CI average of FM_ZF_NS (lowest) significantly differed from FM_ZF (highest) in Term I, FM_NS (lowest) significantly differed from FM_ZF_NS (highest) in Term II, and, finally, FM_NS (lowest) significantly differed from CL (highest) in Term III. At the 20 to 30 cm depth, significant differences among variants were verified solely in Term I. In that case, the CI average of FM_ZF_NS (lowest) significantly differed from FM_ZF (highest), which was the same outcome as for the shallower 10 to 20 cm depth in that term.

**Figure 3:**
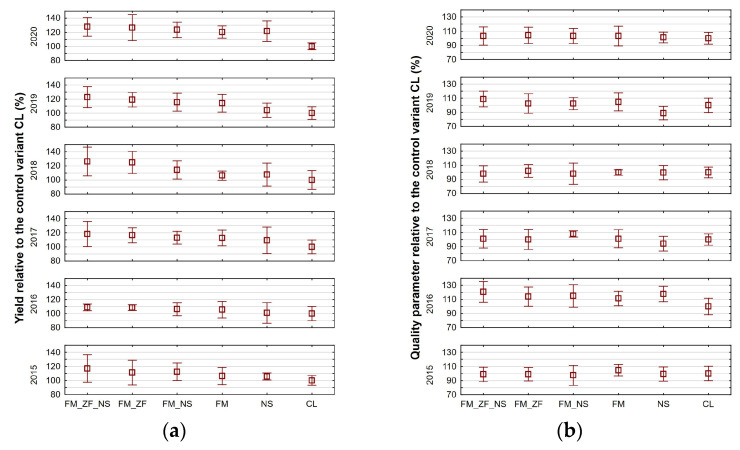
Average values of (**a**) yield and (**b**) given qualitative parameter relative to the respective control variant (CL) for different variants of farmyard manure (FM), farmyard manure stabilized by Z’fix (FM_ZF), and biostimulant NeOsol (NS) application in individual years; vertical bars denote 0.95 confidence intervals.

Figure 3a presents yields of the variants relative to CL for all the years that the experiment was conducted, i.e., from 2015 (Term I) to 2020 (Term III). Their factorial ANOVA proved that there were significant differences among the years as well as among the variants. Concerning the differences among years, the highest yield relative to CL was reached in 2020 (119.896%) and was significantly higher than in all the other years in question. Moreover, the years 2018 (113.177%) and 2019 (112.578%) demonstrated a significant difference compared to the year 2016 (105.048%). In terms of values relative to CL with all the experimental years combined, the variants FM_ZF_NS (120.130%) and FM_ZF (117.780%) significantly differed from FM (110.836%), NS (108.200%), and CL (100.000%). In fact, the latest mentioned CL proved to be significantly lower than all the other variants. With respect to changing crops, comparing absolute values among individual years was not required. One-way ANOVA of absolute values proved that there were significant differences in average yields among variants only in the years 2018, 2019, and 2020 (see Table 3). In 2018, the average yields of FM_ZF_NS (highest) and FM_ZF significantly differed from that attained by CL (lowest). In 2019, FM_ZF_NS reached again the highest yield that significantly differed from NS and CL (lowest). Also, FM_ZF significantly exceeded CL. In 2020, the yields of all five treated variants significantly surpassed the yield produced by CL.

Figure 3b presents the values of the qualitative parameters of the variants relative to CL for all the years that the experiment was conducted. In all cases but one, the qualitative parameter monitored was crude protein content (see Table 3). The factorial ANOVA of relative values proved that there were significant differences among the years only, not among the variants. The qualitative parameter content relative to CL that was reached in 2016 (113.084%) surpassed significantly the value attained in all the other experimental years.

One-way ANOVA of absolute values proved that there were significant differences in qualitative parameter averages among variants only in the years 2016 and 2019 (see Table 3). In 2016, the average crude protein content in winter wheat with FM_ZF_NS (highest) significantly differed from the one attained by CL (lowest). In 2019, FM_ZF_NS reached again the highest average content that, together with the one attained by FM, significantly exceeded NS (lowest).

**Figure 4:**
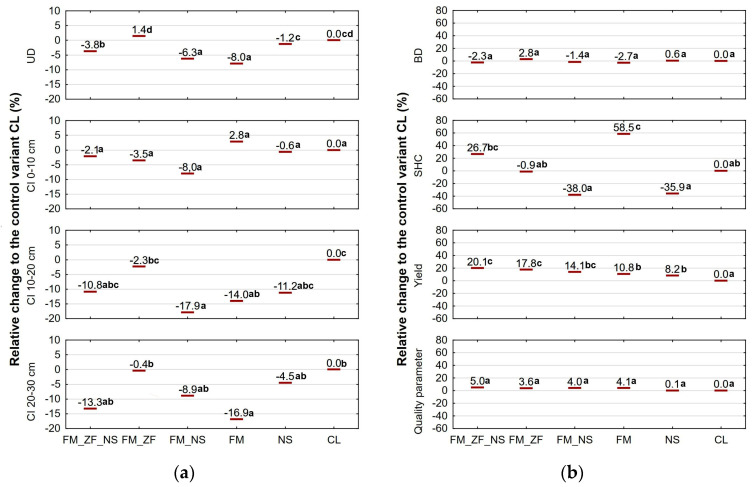
Change in (**a**) soil-related variables (unit draft, cone index) and (**b**) soil- and plant-related variables (bulk density, saturated hydraulic conductivity, yield, qualitative parameters) relative to the respective control variant (CL) for different variants of farmyard manure (FM), farmyard manure stabilized by Z’fix (FM_ZF), and biostimulant NeOsol (NS) application over the monitored period; lowercase letters denote significant differences at probability level of 0.05.

Figure 4 summarises the results of the soil- and plant-related variables in question over the whole monitored period and provides outcomes of the factorial analyses of variance (ANOVA). Concerning UD and CI (see Figure 4a), there were improvements in the case of individual variants except for CI at 0–10 cm of depth. FM (by 8.0%), FM_NS (by 7.3%), and FM_ZF_NS (by 3.8%) had a lower UD than CL. Regarding CI, FM_NS (by 17.9%), and FM (by 14%), they demonstrated lower values than CL at 10–20 cm, and FM was lower again (by 16.9%) at 20–30 cm of depth. With regard to BD and quality parameters (Figure 4b), no substantial differences were found. Concerning SHC (Figure 4b), solely FM (by 58.5%) proved different from CL. On the other hand, the yield (Figure 4b) of all the treated variants, i.e., NS (by 8.2%), FM (by 10.8%), FM_NS (by 14.1%), FM_ZF (by 17.8%), and FM_ZF_NS (by 20.1%), was higher compared to CL.

Table 4 presents the correlation matrix of the variables (BD, UD, SHC, CI, yield, qualitative parameters). Their values relative to the value of the respective control variant (CL) were analysed since, in addition to the variants, there may have been other factors affecting the variables’ absolute values, e.g., soil moisture, machinery passes, and different crops grown. These factors can be considered though to have had the same effect on all the variants including CL. A significantly increasing correlation was verified between BD and UD, between BD and CI at 10–20 cm (BD was measured in the topsoil though), between UD and CI at 10–20 cm, between UD and CI at 20–30 cm, and between CI at 10–20 cm and CI at 20–30 cm. On the contrary, a significantly decreasing correlation was confirmed between BD and SHC, between BD and yield, between BD and quality parameters, between CI at 10–20 cm and yield, and between CI at 20–30 cm and yield. In other words, higher bulk density may have adversely affected unit draft, saturated hydraulic conductivity, quality parameters, and yield. The latter was negatively influenced by the cone index at 10–30 cm of depth.

Table S1 (see Supplementary Materials) presents the values of vegetation indices: (a) NDVI, (b) MSI, and (c) GCI. The images originated from Sentinel-2 satellites using the Google Earth Engine platform, where cloud cover did not exceed 10%. Each experimental variant consisted of 92 pixels. These indices provide spatial information on crop conditions in terms of (a) plant health, (b) water content, and (c) chlorophyll content. In addition, the table contains the one-way ANOVA results for each date separately.

From the results of the vegetation indices, the interconnectedness of water stress and plant health can be observed, as the MSI and NDVI results often correspond with each other. The better values for the CL variant at the beginning of the experiment in 2015 may be attributed to later observation dates in terms of the vegetative period. In the 2018 season, the best health status was reached by the FM variant, where the lowest water stress was also observed. In the 2019 season, it was the FM_ZF_NS variant that achieved high NDVI and low MSI values on most dates, while other variants were rated the highest as far as the chlorophyll content according to the GCI index. Regarding the GCI index, the FM_ZF_NS variant reached often the highest values, which corresponded with its yields having been also the highest (see Table 3).

## 3. Discussion

Numerous studies have demonstrated a correlation between soil organic matter content [23,24] or organic amendments [25,26] and bulk density [24]. According to the USDA, the ideal bulk density for crop growth of silt loam soils is <1.4 g cm^−3^, the root growth is adversely affected at the level of 1.55 g cm^−3^, and root growth is restricted at the level >1.65 g cm^−3^ [27]. In this respect, root growth was not restricted throughout the experiment. In Terms II and III, all variants reached the ideal bulk density, except for the CL variant in Term III (see Table 1). The lowest BD values were achieved in Terms II and III for the FM variant, which was in line with the results of Yu et al., who found a reduction in soil bulk density after cow manure application [28]. The FM_ZF variant reached higher values than FM alone; this finding was supported by the results of Čermáková et al. [29]. The significant reduction in BD between Term I and the following terms indicated a positive effect of long-term application of organic matter to soil, as confirmed by Fu et al. [30].

Tillage is one of the most energy-intensive operations in agriculture [31]; high amounts of energy are devoted to cutting, breaking, and possibly overturning soil layers, reducing the size of clods and rearranging soil aggregates [32]. The results of this study for the NS variant, namely the 1.2% reduction in UD (see Table 1), though not significant, support the findings of the study by Urbanovičová et al. [33], who reported that biostimulants had a positive effect on UD reduction. In that study, UD was reported to decrease by 5.71% when the biostimulant was applied. The decrease in UD with biostimulant treatment was also confirmed by the results of a study conducted by Tuba et al. [12], who reported a 9% reduction in UD in the first year after application. The FM_ZF variant had overall higher UD values than the FM variant; this finding is not consistent with the authors’ previous study [18]. The combination of stabilized manure and biostimulant led to a 3.8% reduction in UD compared to CL. This finding is consistent with a previous study by Šařec et al. [34], where a reduction in UD was also observed after a four-year experiment. Overall, the FM variant presented the highest reduction in UD, which was consistent with a study [35] where plough draft was reduced by 27–38% after eight years of manure application, although the reduction was not as high in our study. The effect of organic matter addition on the reduction in the plough draft was also confirmed in another study [36]. In our study, UD was reduced by almost 8% for the FM variant compared to CL. This reduction could lead to fuel savings of approximately 0.8 L ha^−1^ (assuming an average power delivery efficiency of around 50% and a fuel demand for soil tillage at the level of 20 L ha^−1^). In terms of sustainability, it is necessary to consider the high energy requirements of tillage not only from the agronomic perspective but also from the economic and ecological ones [37]. The carbon dioxide (CO_2_) emitted by the combustion of one litre of diesel oil is approximately equivalent to 2.6–2.8 kg [38,39]; an additional 0.5 kg of CO_2_ is emitted to produce and distribute this one litre of diesel oil. In total, an emission rate of around 3.2 kg of CO_2_ per litre of combusted diesel oil can be assumed [40]. For the FM variant, the CO_2_ emissions coming from diesel combustion are therefore 5.12 kg ha^−1^ lower. Although the reduction in CO_2_ emissions can be ensured by reduced tillage [41], it is necessary to address this issue also in the case of conventional technology. CO_2_ emissions in agriculture, however, are a very complex issue that needs to be considered in a broad ecosystem cycle.

The cone index (CI), also often known as penetration resistance, is considered one of the crucial indicators of soil compaction [42]. Cone index values between 2 and 3 MPa, measured at field capacity, are considered upper limits for root growth [43,44,45]. Exceeding these values can lead to a restriction in root growth and a reduction in plant biomass and yield [46]. CI values up to the depth of 30 cm did not exceed 2.5 MPa (see Table 2). From this point of view, there was no significant restriction of root growth. Manure treated with a stabilizer reached lower values in the upper soil layer than untreated manure, this finding is in accordance with the results of previous studies [18,29]. Celik et al. [47] reported a positive influence of organic amendments on CI; this was confirmed by our results in depth zones of 10–20 cm and 20–30 cm, where FM yielded 14% lower values compared to CL. Tuba et al. [12] reported a 17–23% reduction in penetration resistance when the biostimulant was used; our results did not confirm this finding.

Saturated hydraulic conductivity (SHC) is an important soil hydraulic parameter that affects water flow and dissolved solute transport [48]. SHC is strongly correlated with the soil’s physical properties [49] and can be used as an indicator of water erosion [50]. This issue is currently very significant, as soil loss due to water erosion is expected to increase by 13–22.5% in the EU and UK by 2050 [51]. According to estimates by the Czech Ministry of Agriculture, water erosion causes a total damage of CZK 4.3 billion annually in the Czech Republic. This figure represents solely soil loss and does not include any other damages, e.g., to properties [52]. The SHC values can present a relatively large variability for the Simplified Falling Head Method, especially when a smaller ring diameter is used [53]. However, all outliers in our study were excluded based on Dixon’s Q test. The significant overall increase in SHC over the years suggests a positive effect of field management on soil infiltration (Table 1). Despite the large variability in results, very similar SHC values were measured for the FM and FM_ZF variants in Terms II and III. The positive effect of manure on SHC was reported by Miller et al. [54], who found an increase in SHC from 76 to 128%. This is in accordance with the findings of our study, where there was an increase of 77% in Term II and 308% in Term III. However, treatment of manure with a stabilizer did not lead to an increase in SHC compared to untreated manure, which is inconsistent with the authors’ previous findings [18]. The use of the biostimulant did not lead to an improvement in SHC, which is in accordance with the results of the previous study [55].

Currently, it is necessary to increase crop yields in order to ensure food security related to the demands and needs of a growing population [56]. While organic fertilizer application improves soil health [24,57], the inappropriate use of industrial fertilizers, especially nitrogen and phosphate fertilizers, has a harmful effect on soil health and soil-related ecosystem services [58]. The application of the biostimulant resulted in a higher yield each year compared to the CL variant, though significantly in 2018 and 2019 only (Table 3). This result was consistent with the study by Sulewska et al. [59], who found that phosphorus–potassium fertilization could be replaced with a biostimulator without yield loss. Manure treatment with stabilizer resulted in increased yield each year compared to untreated manure, though again significantly in 2018 and 2019 only. This result was consistent with the study [18], where an increase in sugar beet yield was reported, and with another study [60], where an increase in silage maize yield was observed. The increasing yields of the treated variants compared to CL over the years could be attributed to an extent to an improvement in the soil environment.

Fertilization using manure, whether treated or not, led to an increase in quality parameters, though significantly in 2016 and 2019 only (Table 3). This finding is consistent with studies that show that fertilizer management affects the crude protein content of maize [61,62,63], winter wheat [64,65], and barley [66]. Nitrogen application also influenced crude fat content in canola [67]. Sulewska et al. [68] noted that biostimulant application led to a similar fat content in dry weight of rape seeds as fertilization with conventional P and K. This was in line with the results of our study, where no significant difference was proven between NS and CL variants. Zielewicz et al. [69] observed a 7% increase in crude protein in ryegrass sward when a biostimulant was used, but no evidence for this increase was found in our results, although the crops were different. In addition, Możdżer et al. [70] found that treatment of slurry with a stabilizer and PK fertilization increased the macro-elements’ concentrations in winter rape and spring wheat grain; however, the significant qualitative parameter improvement was not confirmed in our results when the stabilizer was used.

Remote sensing data provide a cheap and relatively accurate source of information that can be used directly in agricultural practice. However, augmenting these data with other in situ data alleviates correct interpretation. In this respect, for instance, an interesting relation could be observed between the SHC values and MSI index in Term I (Table S1), where the lowest water stress was achieved by the CL variant, which presented the highest SHC. In Term II, the variant with the highest SHC had the highest GCI, suggesting the highest chlorophyll content. The results of other researchers indicated a positive effect of the biostimulant on chlorophyll content [14,16,71,72]. These findings partially agree with our results, where the NS variant achieved higher GCI values on most dates compared to the CL variant.

## 4. Materials and Methods

### 4.1. Biostimulant NeOsol

NeOsol, which the Olmix Group produces in Brégan, France, is a granular biostimulator designed to enhance essential soil functions. Utilizing the patented Mineral Inducer Process (MIP) technology, this soil amendment uses the bioactive properties of minerals and specific trace elements to activate biological reactions in the soil. Crucial ingredients such as iron, manganese, copper, and boron stimulate enzymatic reactions essential for the decomposition of raw organic matter, particularly aiding humification processes (e.g., α-glucosidase, β-glucosidase, etc.). The NeOsol complements the MIP technology with SEAweed DRY algae extracts, which provide rich nutrients that stimulate the soil biota.

The composition of this biostimulant is reported to include 28.0% *w*/*w* of CaO, 17.0% *w*/*w* of MgO, and 98.9% *w*/*w* of dry matter, with combustible substances constituting 7% w/DMw. With a pH ranging from 8 to 10, it is highly alkaline.

Depending on the specific crop and local soil conditions, the recommended dosage varies between 110 and 220 kg ha^−1^. NeOsol is typically applied post-harvest and is spread on the soil surface.

### 4.2. Stabilizer Z’fix

Z’fix, which the Olmix Group produces in Brégan, France, is used as an activator of biological transformation in stables, aiming to improve the quality of bedding by controlling the fermentation process of organic matter. The main emphasis lies in improving animal welfare. In addition, the manufacturer mentions a secondary benefit: its ingredients control fermentation in organic matter to preserve the maximum amount of fertilising elements. Z’fix is produced in the form of granules that contain calcium and magnesium carbonates, together with a mixture of micro- and macro-elements such as potassium, sodium, sulphur, iron, and manganese. These ingredients are designed to regulate the fermentation processes in manure, slurry, and compost. The Z’fix also uses MIP technology. Table 5 shows the selected chemical parameters of treated and non-treated FM. The FM used originated from cattle stabled on deep straw bedding. Z’fix was applied at the rate of 1 kg head^−1^ week^−1^ directly to deep bedding.

The composition of Z’fix is organic matter—5% *w*/*w*, CaO—37.5% *w*/*w*, MgO—4.4% *w*/*w*, Na_2_O—3.9% *w*/*w*, SO_3_—0.7% *w*/*w*, K_2_O—0.5% *w*/*w*, P_2_O_5_—0.1% *w*/*w*, Fe—2000 ppm, Mn—150 ppm, and Zn—30 ppm.

### 4.3. Site and Crop Management

The six-year field experiment was conducted near the town of Lázně Bohdaneč in the Královéhradecký region of the Czech Republic (50°27′15.2″ N 15°34′12.5″ E, 365 masl.). According to the Czech Hydrometeorological Institute, this site is located in an area with an annual average temperature of 8.2 °C and an annual rainfall of 732 mm (normal between 1991 and 2020). Table 6 represents the site’s soil conditions at the beginning of the experiment. The experiment was performed on high-quality agricultural soil, namely Modal Luvisol, which, according to the United States Department of Agriculture (USDA) texture triangle, belongs to the silt loam category.

Experimental field of the agricultural company ZEPO Bělohrad a.s. contained six plots, i.e., variants differing by fertilizer and biostimulant applied: farmyard manure stabilized by Z’fix and combined with biostimulant NeOsol (FM_ZF_NS); farmyard manure stabilized by Z’fix (FM_ZF); conventional farmyard manure combined with NeOsol (FM_NS); farmyard manure (FM); biostimulant NeOsol (NL); and a control variant (CL), where only NPK fertilizer was applied at the rate according to the farm standards, respecting crop requirements. In addition to CL, the NPK fertilizer was applied also within the other variants at a rate securing the same overall nitrogen dose., e.g., in the case of NL, the NPK rate did not differ from CL; when complementing farmyard manure, the NPK rate calculated acknowledged manure’s multi-year decomposition. Each variant plot was a rectangle of 30 m × 300 m (0.9 ha), and the positioning on the experimental site was chosen with respect to the headlands. Table 7 provides information on crop rotation and application rates of farmyard manure, both stabilized by Z’fix and conventional manure, and of the biostimulant NeOsol for the respective variants. For the purpose of the article, soil physical characteristics were assessed each season (term) following the autumn manure application, i.e., in the years 2015, 2017, and 2020 (see Table 7).

### 4.4. Data Acquisition and Processing

In situ measurements were always considered for the years following manure applications, i.e., Term I (2015), Term II (2017), and Term III (2020). With regard to the more homogenous moisture distribution in the soil profile, the following variables were measured in spring: cone index, bulk density, and saturated hydraulic conductivity.

Cone index (CI) is used as a crucial indicator of pedocompaction. Its advantage is the relatively rapid and uncomplicated measurement over the entire soil profile—depending on the moisture content. CI is, in fact, a measurement of the soil’s resistance against a cone with a well-defined geometric characteristic (angle, area). The PN70 penetrometer used in this study was developed at the Czech University of Life Sciences Prague (Prague, the Czech Republic). This tailor-made instrument fulfils all the requirements of the ASAE S313.3—the American Society of Agricultural and Biological Engineers (ASABE) agricultural standard. CI was measured in ten repetitions per variant.

Furthermore, bulk density (BD) was determined as another indicator of soil compaction since it can influence all the key soil processes (infiltration, rooting depth, available water capacity, soil porosity, availability of nutrients, activity of soil micro-organisms, etc.) and the productivity of the soil. A soil sample ring kit (Eijkelkamp, Giesbeek, Zevenaar, The Netherlands), where the volume of each ring was 100 cm^3^, was used to collect undisturbed soil samples. BD was sampled in six repetitions per variant, in the topsoil (0–5 cm), without the soil crust—if present. Subsequent analysis of the BD soil samples was carried out in the laboratories of the Faculty of Engineering, Czech University of Life Sciences Prague, according to the national standard CSN EN ISO 17892-2 [73].

**Figure 5:**
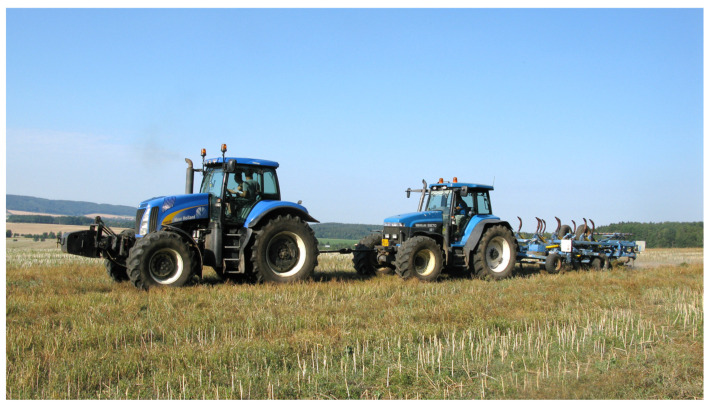
Unit draft (UD) measurement using the towing tractor New Holland T8030 and the implement Farmet Hurikán tine cultivator.

The unit draft (UD) of a tillage implement was measured in autumn—after the crop was harvested. The method of one tractor towing another tractor using a bar equipped with a dynamometer, particularly with strain gauges S-38/200 kN/(LUKAS, Prague, Czech Republic), was used. The towed tractor was mounted with the implement. The towing bar required a horizontal positioning between the two tractors, and it was essential that the working speed and working depth were as constant as possible. In the case of this method, several passes of the above-described gang of machines per one variant were necessary. The passes differed by the implement either working (at least four passes per variant) or not working (at least two passes per variant). In the first case, the dynamometer measured the overall draft of the towed tractor and the working implement, i.e., the overall rolling resistance of the towed tractor and the implement and the implement’s functional draft. In the latter case of the implement not working, the dynamometer measured only the overall rolling resistance of the towed tractor and the implement. Their difference thus provided the value of a functional draft of the implementation. Also, the direction of passes, i.e., potential slope-induced downhill and uphill movement, had to be allowed for. Otherwise, the functional draft might have been biased by the tangential component of the machinery’s gravity. After each pass, the working depth of the implement was measured at both sides of the working width in order to enable unit draft calculation. The tillage depth was set to the maximum depth that the traction conditions and the engine power allowed. The tillage depth was 12.2 ± 0.7 cm in Term I, 22.4 ± 1.2 cm in Term II, and 13.6 ± 1.0 cm in Term III. In all three terms, the Farmet Hurikán tine cultivator was used—in Terms I and III, the towing tractor was a New Holland T8030 (see Figure 5), and in Term II, it was a John Deere 8320R. An NI CompactRIO controller (National Instruments Corporation, Austin, TX, USA) with relevant modules including GPS was employed for data acquisition with the frequency set to 0.1 s. Trimble Business Center 5.70 (Trimble, Westminster, CO, USA) appointed attained data to specific experimental variants.

Saturated hydraulic conductivity (SHC) was determined by a Simplified Falling-Head Technique described by Bagarello et al. [45]. This method is rapid, cost-effective, and does not demand as much water as others. The SHC was measured in fifteen repetitions per variant. Circular infiltrometers (in this study, 0.15 m diameter) and a known quantity of water (0.5 L) were used. The water was poured on the soil surface in the area of the infiltrometer, and the time of infiltration was measured. Before and after the water application, volumetric soil moisture was measured using a Theta Probe (Delta-T Devices, Ltd., Cambridge, UK).

The above-mentioned variables were used for the subsequent calculation of the SHC, resp. the *K_fs_* value (Equation (1)):

(1) $$K_{fs}=\frac{\left(\Delta \theta \right)}{(1-\Delta \theta )t_{\alpha }}\left[\frac{D}{\Delta \theta }-\frac{\left(D+\frac{1}{\alpha^{*}}\right)}{\left(1-\Delta \theta \right)}\ln\left(1+\frac{\left(1-\Delta \theta \right)D}{\left(\Delta \theta \right)(D+\frac{1}{\alpha^{*}})\;}\right)\right],$$

where Δ*θ* = difference between initial soil moisture content and saturated soil moisture content; *D* = the ratio of V (volume of water) and A (area of a cylinder), which is the water level corresponding to the water volume; *t_α_* = infiltration time; and α* = constant according to Elrick et al. [74]; in this study, α* = 12 m^−1^, which is appropriate for the majority of structured soils.

### 4.5. Crop Status

The European Space Agency’s Sentinel-2 satellites were used to acquire the remote sensing data. Their use is recommended for agricultural practice due to the free high-resolution and multi-spectral data (10 m per px) [75]. The data were obtained using Google Earth Engine (Google LLC, San Francisco, CA, USA) and subsequently processed using qGIS (Open Source Geospatial Foundation, Beaverton, OR, USA) and STATISTICA (TIBCO, Palo Alto, CA, USA).

Vegetation status was evaluated using the following indices: the Normalized Difference Vegetation Index (NDVI) [76], one of the most common vegetation indices used for local-scale management purposes as a direct indicator of plant health and growth [77,78,79]; the Moisture Stress Index (MSI) [80], which is highly sensitive to moisture stress in plants and correlates with soil moisture [81]; and the Green Chlorophyll Index (GCI) [82], which is used to estimate the chlorophyll content of various plant species. The chlorophyll content reflects the physiological condition of vegetation; thus, the GCI can be used as an indicator of vegetation health [83]. Comprehensive results of remote sensing observations can be found in Supplementary Materials Table S1.

Grain yield was based on three combine harvester passes per variant. The grain weight of each pass was determined using a trailer positioned on a DINI ARGEO WWSB 16t portable static axle scale (DINI ARGEO S.r.l., Modena, Italy). Mix samples for qualitative analysis were taken from each crossing (three repetitions per variant) and subsequently analysed in laboratories.

Data were processed using MS Excel (Microsoft Corp., Redmond, WA, USA), MS Access (Microsoft Corp., Redmond, WA, USA), and STATISTICA 14 (TIBCO, Palo Alto, CA, USA). Significant differences in the above-mentioned variables among investigated variants were determined through factorial (factors: variant; term) analyses of variance (ANOVA) and Tukey’s HSD post hoc tests with a 95% confidence interval. Correlation analysis of the examined variables (BD, UD, SHC, CI, yield, qualitative parameters) was performed at a probability level of 0.05.

## 5. Conclusions

This study aimed to verify the effect of the biostimulant NeOsol (NS), the manure stabilizer Z’fix (ZF), farmyard manure (FM), and their combinations in real-life conditions of agricultural practice in a six-year experiment. First of all, these treated variants were supposed to reduce soil bulk density (BD), unit draft (UD), and cone index (CI). On the other hand, they should have increased saturated hydraulic conductivity (SHC), yield, and quality parameters. When all three treatments were joined, i.e., farmyard manure stabilised by Z’fix and combined with NeOsol (FM_ZF_NS), only UD decreased significantly and yield increased. The variant of farmyard manure stabilised by Z’fix (FM_ZF) proved solely to increase yield. Conventional farmyard manure combined with NeOsol (FM_NS) significantly decreased UD and CI at 10–20 cm and increased yield. Farmyard manure alone (FM) significantly reduced UD and CI at 10–20 cm and at 20–30 cm. On the contrary, SHC and yield augmented significantly with conventional farmyard manure application. The application of NeOsol significantly raised yield alone. None of the examined treatments proved any adverse effect either on soil or on plant-related variables. Additionally, they are likely to have improved other variables that were not monitored, e.g., ammonia emissions in animal housing, plant canopy cover, etc. A more environmentally sustainable agricultural system is unlikely to be harmful to the environment; it rather generates benefits and protects the land against possible risks.

## Figures and Tables

**Table 1:** Means and standard deviations of soil bulk density (BD), unit draft (UD), and saturated hydraulic conductivity (SHC) for different variants of farmyard manure (FM), farmyard manure stabilized by Z’fix (FM_ZF), and biostimulant NeOsol (NS) application in individual terms; lowercase letters denote significant differences at probability level of 0.05 within each term separately; numbers in bold indicate the most favourable value.

Variable	Variant	Term I	Term II	Term III
Bulk density (g·cm^−3^)	FM_ZF_NS	1.517 ^ab^ ± 0.025	**1.285 ^a^ ± 0.011**	**1.298 ^a^ ± 0.126**
	FM_ZF	1.553 ^b^ ± 0.035	1.374 ^a^ ± 0.011	1.382 ^a^ ± 0.053
	FM_NS	1.483 ^ab^ ± 0.042	1.327 ^a^ ± 0.062	1.323 ^a^ ± 0.091
	FM	1.457 ^a^ ± 0.021	1.304 ^a^ ± 0.025	1.318 ^a^ ± 0.019
	NS	1.487 ^ab^ ± 0.012	1.349 ^a^ ± 0.069	1.381 ^a^ ± 0.020
	CL	**1.450 ^a^ ± 0.026**	1.327 ^a^ ± 0.024	1.415 ^a^ ± 0.026
Unit Draft (kN·m^−2^)	FM_ZF_NS	95.1950 ^b^ ± 10.508	38.662 ^b^ ± 4.815	87.699 ^b^ ± 8.954
	FM_ZF	105.784 ^c^ ± 7.377	36.538 ^a^ ± 3.337	93.372 ^c^ ± 10.549
	FM_NS	**88.856** **^a^ ± 6.662**	38.730 ^b^ ± 4.642	86.271 ^b^ ± 6.012
	FM	94.670 ^b^ ± 8.706	**36.089 ^a^ ± 4.764**	**81.004 ^a^ ± 6.764**
	NS	96.625 ^b^ ± 13.554	37.882 ^ab^ ± 2.809	94.208 ^c^ ± 7.613
	CL	97.755 ^b^ ± 7.852	38.773 ^b^ ± 3.607	94.812 ^c^ ± 6.999
Saturated hydraulic conductivity (mm·h^−1^)	FM_ZF_NS	5.324 ^ab^ ± 3.481	**72.178 ^b^ ± 35.344**	18.816 ^a^ ± 21.883
	FM_ZF	5.451 ^ab^ ± 2.829	25.238 ^a^ ± 17.725	25.033 ^a^ ± 20.985
	FM_NS	3.355 ^a^ ± 1.889	31.189 ^a^ ± 21.198	6.546 ^a^ ± 3.372
	FM	5.589 ^ab^ ± 2.848	52.283 ^ab^ ± 37.831	**52.630 ^b^ ± 26.385**
	NS	3.859 ^a^ ± 1.703	15.206 ^a^ ± 11.374	15.736 ^a^ ± 8.318
	CL	**6.748 ^b^ ± 3.008**	29.555 ^a^ ± 42.198	17.080 ^a^ ± 15.432

**Table 2:** Means and standard deviations of cone index (CI) for different variants of farmyard manure (FM), farmyard manure stabilized by Z’fix (FM_ZF), biostimulant NeOsol (NS), and control variant (CL) application in individual terms; lowercase letters denote significant differences at probability level of 0.05 within each term separately, numbers in bold indicate the most favourable value.

Depth Zone	Variant	Cone Index (MPa)		
		Term I	Term II	Term III
0–10 cm	FM_ZF_NS	0.515 ^a^ ± 0.118	1.040 ^a^ ± 0.338	0.380 ^a^ ± 0.083
	FM_ZF	0.590 ^a^ ± 0.168	0.950 ^a^ ± 0.228	**0.345** **^a^ ± 0.110**
	FM_NS	**0.525** **^a^ ± 0.177**	**0.885** **^a^ ± 0.243**	0.363 ^a^ ± 0.076
	FM	0.605 ^a^ ± 0.161	0.970 ^a^ ± 0.236	0.400 ^a^ ± 0.103
	NS	0.550 ^a^ ± 0.161	0.965 ^a^ ± 0.281	0.400 ^a^ ± 0.086
	CL	0.550 ^a^ ± 0.254	1.040 ^a^ ± 0.241	0.380 ^a^ ± 0.062
10–20 cm	FM_ZF_NS	**1.043** **^a^ ± 0.358**	1.403 ^b^ ± 0.289	0.727 ^ab^ ± 0.328
	FM_ZF	1.437 ^b^ ± 0.466	1.270 ^ab^ ± 0.184	0.770 ^ab^ ± 0.518
	FM_NS	1.140 ^ab^ ± 0.358	**1.197** **^a^ ± 0.248**	**0.593** **^a^ ± 0.319**
	FM	1.237 ^ab^ ± 0.381	1.223 ^ab^ ± 0.290	0.627 ^ab^ ± 0.236
	NS	1.253 ^ab^ ± 0.436	1.303 ^ab^ ± 0.247	0.637 ^ab^ ± 0.346
	CL	1.280 ^ab^ ± 0.445	1.330 ^ab^ ± 0.260	0.903 ^b^ ± 0.495
20–30 cm	FM_ZF_NS	**1.985 ^a^ ± 0.631**	2.055 ^a^ ± 0.551	**1.430 ^a^ ± 0.571**
	FM_ZF	2.575 ^b^ ± 0.588	1.875 ^a^ ± 0.430	1.860 ^a^ ± 0.737
	FM_NS	2.210 ^ab^ ± 0.412	1.930 ^a^ ± 0.542	1.611 ^a^ ± 0.768
	FM	2.185 ^ab^ ± 0.485	**1.605 ^a^ ± 0.454**	1.490 ^a^ ± 0.620
	NS	2.485 ^ab^ ± 0.712	1.915 ^a^ ± 0.382	1.665 ^a^ ± 0.595
	CL	2.445 ^ab^ ± 0.717	2.030 ^a^ ± 0.591	1.840 ^a^ ± 0.426

**Table 3:** Means and standard deviations of yield from standard dry matter (DM) content and of a given qualitative parameter for different variants of farmyard manure (FM), farmyard manure stabilized by Z’fix (FM_ZF), biostimulant NeOsol (NS), and control variant (CL) application in individual years; lowercase letters denote significant differences at probability level of 0.05 within each year separately, numbers in bold indicate the most favourable value..

Year:	2015 Term I	2016	2017 Term II	2018	2019	2020 Term III
Crop:	Silage maize	Winter wheat	Winter barley	Oilseed rape	Winter wheat	Winter wheat
Standard moisture (% DM):	38.0	14.5	14.5	9.0	14.5	14.5
Variant	Yield at standard moisture (t ha^−1^)					
FM_ZF_NS	**35.200 ^a^ ± 3.681**	**7.870 ^a^ ± 0.214**	**7.780 ^a^ ± 0.723**	**4.040 ^b^ ± 0.412**	**7.860 ^c^ ± 0.602**	**7.380 ^b^ ± 0.478**
FM_ZF	33.500 ^a^ ± 3.341	7.850 ^a^ ± 0.188	7.660 ^a^ ± 0.440	3.990 ^b^ ± 0.314	7.620 ^bc^ ± 0.414	7.320 ^b^ ± 0.672
FM_NS	33.800 ^a^ ± 2.356	7.680 ^a^ ± 0.415	7.440 ^a^ ± 0.381	3.660 ^ab^ ± 0.259	7.390 ^abc^ ± 0.517	7.130 ^b^ ± 0.406
FM	32.000 ^a^ ± 2.314	7.630 ^a^ ± 0.540	7.410 ^a^ ± 0.458	3.400 ^ab^ ± 0.130	7.300 ^abc^ ± 0.509	6.950 ^b^ ± 0.319
NS	31.800 ^a^ ± 0.935	7.310 ^a^ ± 0.666	7.200 ^a^ ± 0.776	3.440 ^ab^ ± 0.326	6.660 ^ab^ ± 0.410	7.020 ^b^ ± 0.533
CL	30.100 ^a^ ± 1.289	7.230 ^a^ ± 0.456	6.580 ^a^ ± 0.403	3.200 ^a^ ± 0.264	6.400 ^a^ ± 0.363	5.780 ^a^ ± 0.174
	Qualitative parameter and its value (% DM)					
	Crude protein	Crude protein	Crude protein	Crude fat	Crude protein	Crude protein
FM_ZF_NS	8.472 ^a^ ± 0.548	**12.900 ^b^ ± 0.987**	10.200 ^a^ ± 0.829	43.000 ^a^ ± 3.178	**13.600 ^b^ ± 0.876**	9.700 ^a^ ± 0.769
FM_ZF	8.493 ^a^ ± 0.510	12.200 ^ab^ ± 0.912	10.100 ^a^ ± 0.896	**44.830 ^a^ ± 2.480**	12.800 ^ab^ ± 1.087	**9.800 ^a^ ± 0.685**
FM_NS	8.361 ^a^ ± 0.763	12.300 ^ab^ ± 1.082	**10.900 ^a^ ± 0.273**	43.120 ^a^ ± 4.146	12.800 ^ab^ ± 0.675	9.700 ^a^ ± 0.628
FM	**8.976 ^a^ ± 0.438**	11.900 ^ab^ ± 0.697	10.200 ^a^ ± 0.809	43.920 ^a^ ± 1.067	13.100 ^b^ ± 1.005	9.700 ^a^ ± 0.823
NS	8.509 ^a^ ± 0.542	12.600 ^ab^ ± 0.737	9.500 ^a^ ± 0.671	43.770 ^a^ ± 2.771	11.100 ^a^ ± 0.752	9.500 ^a^ ± 0.747
CL	8.575 ^a^ ± 0.545	10.700 ^a^ ± 0.782	10.100 ^a^ ± 0.512	43.990 ^a^ ± 2.103	12.500 ^ab^ ± 0.798	9.400 ^a^ ± 0.494

**Table 4:** Correlation matrix of Pearson correlation coefficients (r) and probabilities (*p*) for soil bulk density (BD), unit draft (UD), saturated hydraulic conductivity (SHC), cone index (CI) at three depth zones, yield, and quality parameters; * denotes significant differences at probability level of 0.05.

	BD	UD	SHC	CI at			Yield	Quality
				0–10 cm	10–20 cm	0–30 cm		Parameter
BD		r = 0.6008 *	r = −0.4802 *	r = −0.0700	r = 0.6366 *	r = 0.4597	r = −0.5221 *	r = −0.4951 *
		*p* = 0.0084	*p* = 0.0437	*p* = 0.783	*p* = 0.0045	*p* = 0.0549	*p* = 0.0263	*p* = 0.0367
UD	r = 0.6008 *		r = −0.3177	r = 0.1233	r = 0.6863 *	r = 0.7554 *	r = −0.4133	r = −0.1499
	*p* = 0.0084		*p* = 0.1989	*p* = 0.6260	*p* = 0.0017	*p* = 0.0003	*p* = 0.0882	*p* = 0.5527
SHC	r = −0.4802 *	r = −0.3177		r = 0.1612	r = −0.1181	r = −0.1903	r = 0.2672	r = 0.3161
	*p* = 0.0437	*p* = 0.1989		*p* = 0.5229	*p* = 0.6407	*p* = 0.4495	*p* = 0.2837	*p* = 0.2013
CI at	r = −0.0700	r = 0.1233	r = 0.1612		r = 0.0899	r = 0.0637	r = −0.1855	r = −0.0579
0–10 cm	*p* = 0.783	*p* = 0.6260	*p* = 0.5229		*p* = 0.7227	*p* = 0.8018	*p* = 0.4612	*p* = 0.8196
10–20 cm	r = 0.6366 *	r = 0.6863 *	r = −0.1181	r = 0.0899		r = 0.6730 *	r = −0.6607 *	r = −0.3527
	*p* = 0.0045	*p* = 0.0017	*p* = 0.6407	*p* = 0.7227		*p* = 0.0022	*p* = 0.0028	*p* = 0.1512
20–30 cm	r = 0.4597	r = 0.7554 *	r = −0.1903	r = 0.0637	r = 0.6730 *		r = −0.4737 *	r = −0.1719
	*p* = 0.0549	*p* = 0.0003	*p* = 0.4495	*p* = 0.8018	*p* = 0.0022		*p* = 0.0470	*p* = 0.4952
Yield	r = −0.5221 *	r = −0.4133	r = 0.2672	r = −0.1855	r = −0.6607 *	r = −0.4737 *		r = 0.3516
	*p* = 0.0263	*p* = 0.0882	*p* = 0.2837	*p* = 0.4612	*p* = 0.0028	*p* = 0.0470		*p* = 0.1525
Quality	r = −0.4951 *	r = −0.1499	r = 0.3161	r = −0.0579	r = −0.3527	r = −0.1719	r = 0.3516	
parameter	*p* = 0.0367	*p* = 0.5527	*p* = 0.2013	*p* = 0.8196	*p* = 0.1512	*p* = 0.4952	*p* = 0.1525	

**Table 5:** Chemical analysis of farmyard manure treated by Z’fix (FM_ZF) and of conventional farmyard manure (FM).

Variant	Dry Matter %	N kg t^−1^	C:N	P_2_O_5_	K_2_O	CaO	MgO	pH
FM_ZF	23	7.1	15.1:1	4.2	7.9	6.44	1.99	9.5
FM	23	5.9	22.3:1	3.5	6.3	4.90	1.60	8.9

**Table 6:** Chemical and physical soil analysis undertaken on 13 August 2014.

	Soil Profile Depth (m)		Unit
	0.00–0.30	0.30–0.65	
Clay (<0.002 mm)	15	21	% *w*/*w*
Silt (0.002–0.05 mm)	66	67	% *w*/*w*
Very fine sand (0.05–0.10 mm)	3	1	% *w*/*w*
Fine sand (0.10–0.25 mm)	16	11	% *w*/*w*
Bulk density	1.56	1.52	g·cm^−3^
Porosity	41.97	42.64	% *w*/*w*
Hummus content	1.81	0.58	% *w*/*w*
Cation exchange capacity	110	120	mmol·kg^−1^
Volumetric moisture	31.5	23.7	% *v*/*v*
pH (KCl)	4.99	5.09	

**Table 7:** Crop rotation and field management.

Season	Crop	Variety	Sowing Date	Harvest Date	Farmyard Manure (t ha^−1^)	NeOsol (kg ha^−1^)
2014	Winter wheat	-	-	-	50	200
2015 Term I	Maize	SY Kairo	27 April 2015	9 September 2015	-	200
2016	Winter wheat	Turandot	7 October 2015	17 August 2016	30	200
2017 Term II	Barley	Titus	20 October 2016	19 July 2017	-	150
2018	Rape seed	Inspiration	15 August 2017	16 July 2018	-	150
2019	Winter wheat	Reform	22 September 2018	9 August 2019	30	150
2020 Term III	Winter wheat	Reform	24 September 2019	9 August 2020	-	150

## Data Availability

The data presented in this study are available from the corresponding author upon reasonable request.

## Supplementary Materials

The following supporting information can be downloaded at: https://www.mdpi.com/article/10.3390/plants13070920/s1, Table S1: Means and standard deviations of vegetation indices: Normalized Difference Vegetation Index (NDVI), Moisture Stress Index (MSI), and Green Chlorophyll Index (GCI) for different variants of farmyard manure (FM), farmyard manure stabilized by Z’fix (FM_ZF), biostimulant NeOsol (NS), and control variant (CL) application in individual dates; lowercase letters denote significant differences at probability level of 0.05 within each date separately.
