# Supplementary material for: Effect of Biostimulant, Manure Stabilizer, and Manure on Soil Physical Properties and Vegetation Status
Source: Plants (Basel). 2024 Mar 22;13(7):920. doi: 10.3390/plants13070920 (PMC11013216; doi:10.3390/plants13070920)
Supplement: Supplementary file 1 [file plants-13-00920-s001.zip › plants-2913712-supplementary.pdf]

Supplementary Materials: Table S1. Means and standard deviations of vegetation indices Normalized Difference Vegetation Index (NDVI), Moisture Stress Index (MSI), and Green Chlorophyll Index (GCI) for different variants of farmyard manure (FM), farmyard manure stabilized by Z'fix (FM_ZF), of biostimulant NeOsol (NS), and control variant (CL) application in individual dates; lower case letters denote significant differences at probability level of 0.05 within each date separately; numbers in bold indicate the most favourable value.

**Date**	**NDVI**						**MSI**						**GCI**					
	**FM_ZF_NS**	**FM_ZF**	**FM_NS**	**FM**	**NS**	**CL**	**FM_ZF_NS**	**FM_ZF**	**FM_NS**	**FM**	**NS**	**CL**	**FM_ZF_NS**	**FM_ZF**	**FM_NS**	**FM**	**NS**	**CL**
11. Jul.	0.573^{d}	0.621^{e}	0.698^{ab}	0.716^{bc}	0.686^{a}	**0.719^{c}**	0.641^{e}	0.569^{d}	0.473^{c}	0.452^{ab}	0.470^{bc}	**0.442^{a}**	**0.641^{e}**	0.569^{d}	0.473^{c}	0.452^{ab}	0.469^{bc}	0.443^{a}
2015	±0.065	±0.072	±0.029	±0.026	±0.040	±0.037	±0.072	±0.076	±0.030	±0.024	±0.033	±0.033	±0.072	±0.076	±0.030	±0.024	±0.033	±0.033
3.Aug.	0.613^{c}	0.647^{d}	0.698^{ab}	**0.707^{a}**	0.683^{b}	**0.707^{a}**	0.574^{c}	0.509^{b}	0.443^{a}	0.435^{a}	0.447^{a}	**0.428^{a}**	**0.574^{c}**	0.509^{b}	0.443^{a}	0.435^{a}	0.447^{a}	0.428^{a}
2015	±0.049	±0.057	±0.029	±0.308	±0.043	±0.039	±0.062	±0.065	±0.032	±0.035	±0.043	±0.039	±0.062	±0.065	±0.032	±0.035	±0.043	±0.039
30. Aug.	0.547^{b}	0.614^{c}	0.661^{a}	0.655^{a}	0.641^{a}	**0.663^{a}**	0.715^{c}	0.614^{b}	0.555^{a}	0.556^{a}	0.561^{a}	**0.540^{a}**	**0.715^{c}**	0.614^{b}	0.555^{a}	0.556^{a}	0.561^{a}	0.540^{a}
2015	±0.090	±0.059	±0.031	±0.038	±0.045	±0.042	±0.106	±0.078	±0.041	±0.046	±0.053	±0.049	±0.106	±0.777	±0.041	±0.458	±0.053	±0.049
17. Mar.	0.336^{b}	0.331^{b}	0.357^{a}	0.359^{a}	0.352^{a}	**0.381^{c}**	0.951^{c}	0.963^{c}	0.926^{ab}	0.931^{a}	0.936^{a}	**0.915^{b}**	0.951^{c}	**0.963^{c}**	0.926^{ab}	0.931^{a}	0.936^{a}	0.915^{b}
2016	±0.026	±0.027	±0.022	±0.034	±0.022	±0.032	±0.035	±0.042	±0.031	±0.039	±0.025	±0.036	±0.035	±0.042	±0.042	±0.039	±0.025	±0.036
27. Mar.	0.405^{b}±	0.392^{c}±	0.427^{a}	**0.431^{a}**	0.407^{b}	0.424^{a}	0.870^{c}	0.888^{d}	0.849^{ab}	0.846^{a}	0.860^{bc}	**0.844^{a}**	0.870^{c}	**0.888^{d}**	0.849^{ab}	0.846^{a}	0.860^{bc}	0.844^{a}
2016	0.025	0.025	±0.023	±0.033	±0.030	±0.028	±0.032	±0.035	±0.030	±0.033	±0.025	±0.026	±0.032	±0.035	±0.030	±0.033	±0.025	±0.026
9. May	**0.762^{c}**	0.723^{a}	0.750^{bc}	0.731^{ab}	0.672^{d}	0.734^{ab}	**0.406^{b}**	0.434^{c}	0.416^{ab}	0.420^{a}	0.467^{d}	0.426^{ac}	0.406^{b}	0.434^{c}	0.416^{ab}	0.420^{a}	**0.466^{d}**	0.426^{ac}
2016	±0.025	±0.038	±0.022	±0.042	±0.082	±0.028	±0.023	±0.018	±0.018	±0.025	±0.037	±0.035	±0.023	±0.018	±0.018	±0.025	±0.037	±0.035
8. Jun.	**0.751^{a}**	0.738^{ab}	0.746^{a}	0.724^{b}	0.677^{c}	0.746^{a}	**0.328^{a}**	0.338^{a}	0.333^{a}	0.372^{b}	0.401^{c}	0.338^{a}	0.328^{a}	0.338^{a}	0.333^{a}	0.372^{b}	**0.401^{c}**	0.338^{a}
2016	±0.024	±0.026	±0.026	±0.041	±0.063	±0.027	±0.018	±0.022	±0.021	±0.032	±0.045	±0.031	±0.018	±0.022	±0.021	±0.032	±0.045	±0.031
11.May	0.879^{c}	0.886^{c}	0.903^{ab}	0.909^{ab}	0.900^{a}	**0.911^{b}**	0.373^{d}	0.358^{c}	0.334^{b}	0.320^{a}	0.319^{a}	**0.313^{a}**	**0.373^{d}**	0.358^{c}	0.334^{b}	0.320^{a}	0.319^{a}	0.313^{a}
2017	±0.023	±0.024	±0.015	±0.019	±0.026	±0.017	±0.031	±0.029	±0.022	±0.025	±0.029	±0.025	±0.031	±0.029	±0.022	±0.025	±0.029	±0.025
3. Jun.	0.866^{c}	0.873^{b}	0.878^{ab}	0.881^{a}	0.878^{ab}	**0.883^{a}**	0.322^{c}	0.308^{b}	0.298^{a}	**0.293^{a}**	0.296^{a}	0.298^{a}	**0.322^{c}**	0.308^{b}	0.298^{a}	0.293^{a}	0.296^{a}	0.298^{a}
2017	±0.015	±0.012	±0.013	±0.016	±0.018	±0.018	±0.020	±0.013	±0.017	±0.020	±0.021	±0.019	±0.020	±0.013	±0.017	±0.020	±0.021	±0.019
20.Jun.	0.726^{a}	0.730^{a}	**0.737^{a}**	0.729^{a}	0.706^{b}	0.698^{b}	0.442^{a}	0.422^{b}	**0.407^{a}**	0.408^{a}	0.412^{ab}	0.423^{b}	**0.442^{c}**	0.422^{b}	0.407^{a}	0.408^{a}	0.412^{ab}	0.423^{b}
2017	±0.036	±0.038	±0.039	±0.041	±0.045	±0.053	±0.027	±0.022	±0.025	±0.030	±0.028	±0.030	±0.027	±0.022	±0.025	±0.030	±0.028	±0.307

28. Sept.	0.601^{a}	0.636^{b}	**0.692^{e}**	0.620^{ab}	0.517^{c}	0.567^{d}	0.674^{a}	0.629^{c}	**0.580^{b}**	0.666^{a}	0.776^{e}	0.730^{d}	0.674^{a}	0.629^{c}	0.580^{b}	0.666^{a}	**0.776^{d}**	0.730^{e}
2017	±0.071	±0.085	±0.060	±0.063	±0.074	±0.089	±0.071	±0.082	±0.064	±0.068	±0.082	±0.098	±0.072	±0.082	±0.064	±0.068	±0.082	±0.098
15 Mar.	0.339^{b}	0.359^{a}	**0.407^{d}**	0.386^{c}	0.352^{ab}	0.367^{a}	0.883^{e}	0.816^{ab}	**0.740^{d}**	0.800^{a}	0.849^{c}	0.843^{bc}	**0.883^{e}**	0.816^{ab}	0.740^{d}	0.780^{a}	0.849^{d}	0.843^{bc}
2018	±0.029	±0.033	±0.028	±0.036	±0.049	±0.049	±0.063	±0.058	±0.049	±0.065	±0.087	±0.079	±0.063	±0.058	±0.049	±0.065	±0.087	±0.079
21. Apr.	0.712^{ab}	0.736^{bc}	**0.799^{d}**	0.759^{c}	0.698^{a}	0.721^{ab}	0.548^{a}	0.476^{b}	**0.413^{c}**	0.457^{b}	0.529^{a}	0.517^{a}	**0.548^{a}**	0.476^{b}	0.413^{c}	0.457^{b}	0.529^{a}	0.517^{a}
2018	±0.072	±0.070	±0.047	±0.060	±0.090	±0.079	±0.088	±0.074	±0.059	±0.079	±0.108	±0.084	±0.088	±0.074	±0.059	±0.079	±0.108	±0.084
31. Oct.	**0.316^{d}**	0.299^{b}	0.296^{b}	0.275^{c}	0.254^{a}	0.253^{a}	**1.120^{c}**	1.153^{d}	1.168^{a}	1.177^{ab}	1.189^{b}	1.203^{e}	1.119^{c}	1.153^{d}	1.168^{a}	1.177^{ab}	1.189^{b}	**1.203^{e}**
2018	±0.020	±0.015	±0.012	±0.013	±0.016	±0.016	±0.034	±0.027	±0.033	±0.033	±0.026	±0.028	±0.034	±0.027	±0.032	±0.033	±0.026	±0.028
15. Nov.	**0.553^{c}**	0.536^{b}	**0.553^{c}**	0.533^{b}	0.499^{a}	0.505^{a}	**0.897^{a}**	0.916^{bc}	0.909^{ab}	0.926^{c}	0.955^{d}	0.952^{d}	0.897^{a}	0.916^{bc}	0.909^{ab}	0.926^{c}	**0.955^{d}**	0.952^{d}
2018	±0.011	±0.016	±0.017	±0.024	±0.031	±0.037	±0.024	±0.023	±0.030	±0.032	±0.039	±0.041	±0.024	±0.023	±0.030	±0.032	±0.039	±0.041
1. Apr.	**0.700^{c}**	0.629^{a}	0.663^{b}	0.663^{b}	0.624^{a}	0.631^{a}	**0.635^{c}**	0.686^{b}	0.674^{a}	0.668^{a}	0.689^{b}	0.707^{d}	0.635^{c}	0.686^{b}	0.674^{a}	0.668^{a}	0.689^{b}	**0.707^{d}**
2019	±0.029	±0.018	±0.027	±0.037	±0.045	±0.049	±0.021	±0.022	±0.024	±0.027	±0.038	±0.040	±0.021	±0.022	±0.024	±0.027	±0.038	±0.040
19. Apr.	**0.743^{b}**	0.681^{c}	0.706^{a}	0.729^{b}	0.703^{a}	0.699^{a}	**0.529^{c}**	0.584^{b}	0.573^{ab}	0.543^{c}	0.561^{a}	0.575^{ab}	0.529^{c}	**0.584^{b}**	0.573^{ab}	0.544^{c}	0.561^{a}	0.575^{ab}
2019	±0.031	±0.024	±0.040	±0.045	±0.047	±0.047	±0.029	±0.024	±0.032	±0.037	±0.042	±0.047	±0.029	±0.024	±0.032	±0.037	±0.042	±0.047
3.Jul.	0.878^{a}	0.870^{c}	0.882^{ab}	**0.885^{b}**	0.876^{a}	0.881^{ab}	0.306^{ab}	0.311^{b}	0.292^{cd}	**0.289^{c}**	0.306^{ab}	0.301^{ad}	0.306^{ab}	**0.311^{b}**	0.292^{cd}	0.289^{c}	0.306^{ab}	0.301^{ad}
2019	±0.011	±0.015	±0.017	±0.015	±0.020	±0.014	±0.018	±0.019	±0.022	±0.023	±0.030	±0.024	±0.018	±0.019	±0.022	±0.023	±0.030	±0.024
31.Oct.	0.157^{a}	0.155^{ab}	0.154^{ab}	0.153^{b}	**0.158^{a}**	0.155^{ab}	**1.290^{b}**	1.315^{a}	1.303^{ab}	1.306^{ab}	1.306^{ab}	1.314^{a}	1.290^{b}	**1.315^{a}**	1.303^{ab}	1.306^{ab}	1.306^{ab}	1.314^{a}
2019	±0.010	±0.009	±0.008	±0.008	±0.009	±0.010	±0.037	±0.041	±0.042	±0.037	±0.035	±0.035	±0.037	±0.041	±0.042	±0.037	±0.035	±0.035
18. Apr.	0.558^{b}	0.566^{b}	0.588^{c}	0.602^{a}	0.601^{a}	**0.608^{a}**	0.764^{c}	0.755^{c}	0.726^{d}	0.710^{b}	0.705^{ab}	**0.699^{a}**	**0.764^{c}**	0.755^{c}	0.726^{d}	0.710^{b}	0.705^{ab}	0.699^{a}
2020	±0.027	±0.018	±0.024	±0.035	±0.037	±0.040	±0.024	±0.015	±0.024	±0.029	±0.023	±0.030	±0.024	±0.015	±0.024	±0.029	±0.023	0.030
28.Apr.	0.550^{ab}	0.544^{a}	0.551^{ab}	0.548^{ab}	0.556^{bc}	**0.567^{c}**	0.575^{ab}	**0.568^{a}**	0.571^{a}	0.603^{b}	0.632^{c}	0.641^{c}	0.575^{ab}	0.568^{a}	0.571^{a}	0.603^{b}	0.632^{c}	**0.641^{c}**
2020	±0.021	±0.019	±0.025	±0.035	±0.035	±0.036	±0.025	±0.031	±0.026	±0.049	±0.100	±0.116	±0.025	±0.031	±0.026	±0.049	±0.100	±0.115
18.May	0.878^{b}	0.878^{b}	0.891^{a}	0.893^{a}	0.890^{a}	**0.896^{a}**	0.365^{c}	0.358^{c}	0.339^{b}	0.331^{a}	0.333^{ab}	**0.324^{d}**	**0.365^{c}**	0.358^{c}	0.339^{b}	0.331^{a}	0.333^{ab}	0.324^{d}
2020	±0.011	±0.010	±0.010	±0.009	±0.017	±0.016	±0.016	±0.014	±0.013	±0.012	±0.025	±0.022	±0.016	±0.014	±0.013	±0.012	±0.025	±0.022
22.Jun.	0.879^{a}	0.882^{a}	0.882^{a}	0.881^{a}	0.882^{a}	**0.887^{b}**	0.322^{e}	0.316^{c}	0.312^{bc}	0.307^{a}	0.310^{ab}	**0.299^{d}**	**0.322^{e}**	0.316^{c}	0.312^{bc}	0.307^{a}	0.309^{ab}	0.299^{d}
2020	±0.006	±0.005	±0.008	±0.013	±0.010	±0.009	±0.009	±0.006	±0.013	±0.017	±0.015	±0.012	±0.009	±0.006	±0.013	±0.017	±0.015	±0.012
